# The US Residency Selection Process After the United States Medical Licensing Examination Step 1 Pass/Fail Change: Overview for Applicants and Educators

**DOI:** 10.2196/37069

**Published:** 2023-01-06

**Authors:** Ahmad Ozair, Vivek Bhat, Donald K E Detchou

**Affiliations:** 1 Miami Cancer Institute Baptist Health South Florida Miami, FL United States; 2 Faculty of Medicine King George’s Medical University Lucknow India; 3 St John's Medical College Bangalore India; 4 Department of Neurosurgery Hospital of the University of Pennsylvania Philadelphia, PA United States; 5 Thomas William Langfitt Neurosurgical Society University of Pennsylvania Perelman School of Medicine Philadelphia, PA United States

**Keywords:** admission, assessment, postgraduate training, selection, standardized testing

## Abstract

The United States Medical Licensing Examination (USMLE) Step 1, arguably the most significant assessment in the USMLE examination series, changed from a 3-digit score to a pass/fail outcome in January 2022. Given the rapidly evolving body of literature on this subject, this paper aims to provide a comprehensive review of the historical context and impact of this change on various stakeholders involved in residency selection. For this, relevant keyword-based searches were performed in PubMed, Google Scholar, and Scopus to identify relevant literature. Given the unique history of USMLE Step 1 in the US residency selection process and the score’s correlation with future performance in board-certifying examinations in different specialties, this scoring change is predicted to significantly impact US Doctor of Medicine students, US Doctor of Osteopathic Medicine students, international medical graduates, and residency program directors, among others. The significance and the rationale of the pass/fail change along with the implications for both residency applicants and educators are also summarized in this paper. Although medical programs, academic institutions, and residency organizing bodies across the United States have swiftly stepped up to ensure a seamless transition and have attempted to ensure equity for all, the conversion process carries considerable uncertainty for residency applicants. For educators, the increasing number of applications conflicts with holistic application screening, leading to the expected greater use of objective measures, with USMLE Step 2 Clinical Knowledge likely becoming the preferred screening tool in lieu of Step 1.

## Introduction

The United States Medical Licensing Examination (USMLE) consists of 3 examinations (USMLE Step 1, Step 2, and Step 3) that medical students/graduates must pass before entering and completing postgraduate clinical residency training in the United States [1]. The USMLE program is jointly administered by the National Board of Medical Examiners (NBME), the Educational Commission for Foreign Medical Graduates (ECFMG), and the Federation of State Medical Boards (FSMB) [2-4]. The USMLE Step 1 tests candidates’ knowledge of the preclinical basic sciences, namely, anatomy, biochemistry, immunology, microbiology, pathology, and pharmacology, while Steps 2 and 3 test candidates’ clinical knowledge. Typically, USMLE Steps 1 and 2 are completed by US students—both MD (Doctor of Medicine) and DO (Doctor of Osteopathic Medicine) candidates during medical school. USMLE Step 2 has historically been composed of 2 components: Step 2 CK (clinical knowledge) and Step 2 Clinical Skills. USMLE Step 3 is typically completed by these students just after medical school graduation or during residency.

For over 16 years, the USMLE Step 1, Step 2 CK, and Step 3 have been criterion-referenced, computer-based assessments. These exams historically provided a 3-digit score, similar to the Medical Council of Canada Qualifying Examination (MCCQE) Part I examination in Canada [5], the National Eligibility cum Entrance Test for Post-Graduation (NEET-PG) in India [6], and the Comprehensive Osteopathic Medical Licensing Examination (COMLEX) of the United States, taken by students of DO schools alongside the USMLE, which all provide numeric scores and percentiles. However, these exams are different from USMLE’s counterparts in the United Kingdom, where the Professional and Linguistic Assessment Board 1 and 2 examinations function as pass/fail-only assessments. Meanwhile, the USMLE Step 2 Clinical Skills exam evaluated candidates through an in-person structured clinical assessment and provided only a pass/fail outcome. However, the latter, first introduced in 2004, was permanently suspended in 2020 due to COVID-19–related restrictions on testing [1]. This change resulted in only 3 tests remaining for candidates aiming to join and complete a residency program in the United States, all providing 3-digit scores for candidates passing them.

In March 2019, the Invitational Conference on USMLE Scoring (InCUS) was held with delegates from 5 major bodies of medical education in the United States—Association of American Medical Colleges, American Medical Association, NBME, FSMB, and ECFMG—with the aim being to “facilitate broader system-wide changes to improve the transition from undergraduate medical education to graduate medical education” [2]. The group, as a consensus, felt that the current system merited wide-spanning changes. In the following year, in 2020, FSMB and NBME announced that score reporting for USMLE Step 1 would change from a 3-digit numeric score to reporting a pass/fail outcome [3,4]. This change finally came into effect on January 26, 2022. Notably, NBME and ECFMG announced that all scores for USMLE Step 1 exams taken prior to the date of change will continue to be reported as the traditional 3-digit score, with no retroactive alteration of transcripts [7]. In a parallel move, the National Board of Osteopathic Medical Examiners announced that COMLEX Level 1—the first of the 3 exams taken by DO candidates as a requirement for osteopathic medicine licensure, as well as medical school graduation, would also transition to a pass/fail reporting system from May 2022 [8].

At the time of writing this paper, less than a year has passed since the scoring change came into effect. Importantly, candidates who had taken and obtained a score on USMLE Step 1 would not have their scores turned to pass/fail at any time in the future. In the US Residency Match Cycle of 2023, which is ongoing at the time of writing, there is a substantial, although unquantified, proportion of candidates with a pass/fail outcome, while several applicants have Step 1 scores. The vast majority of medical students receiving pass/fail reports will likely apply only in the Match Cycle of 2024 and beyond; therefore, definitive implications of this change remain to be seen.

Given the rapidly evolving body of literature on this subject, this paper aims to provide a comprehensive summary of the historical context of this change and the potential impact on various stakeholders involved in residency selection. This paper also aims to review the key studies that have emerged since the pass/fail change was announced to happen. For this, appropriate keyword-based searches were performed in PubMed, Google Scholar, and Scopus in order to identify relevant literature. Empirical data on the impact of this change can only be assessed from literature emerging after the conclusion of Match 2023 and potentially even Match 2024. However, some comprehension may be reached from reviewing the surveys and perspectives coauthored by applicants, program directors, leadership of professional organizations, etc, discussing the potential impact of the change.

## Significance of the USMLE scores

The USMLE was originally intended only for licensure purposes [2]. However, over the years, residency and fellowship programs increasingly co-opted USMLE scores for secondary uses, with these scores gradually becoming one of the most important factors influencing residency selection [9]. According to a 2020 survey by the National Residency Matching Program, 90% of the program directors considered candidates’ USMLE Step 1 score while deciding whether to invite them for an interview, with 55% reporting that they had a target score for candidates, implying the use of Step 1 as a screening tool [9]. The reliance on USMLE Step 1 scores for residency application considerations was particularly notable in competitive specialties. A case in point is a survey of over half of all neurosurgical residency program directors that found that 77% of them had always screened candidates using Step 1 scores [10], and a score of >245 was the most significant predictor of success in the neurosurgery match (1990-2007) [11]. Thus, aspirants for these specialties would find their specialty of choice out of reach if they had a low Step 1 score. In addition to residency selection, Step 1 scores were utilized for selection into honor societies and away rotations, which also influence, albeit to a lesser extent, the residency selection.

Performance in the Step 1 examination was also known to be widely correlated with performance on in-training exams taken during residency and with board certification passing rates, as demonstrated by a large amount of published literature across numerous specialties. For instance, Swanson et al [12] reported in 2009 that orthopedic surgery residents having low scores on Step 1 and Step 2 CK were at significantly higher risk of failing the Part I of the American Board of Orthopedic Surgery Certifying Examination. Similarly, in 2010, Dougherty et al [13] reported that Step 1 scores correlated with American Board of Orthopedic Surgery Part 1 scores and commented that it may continue to be used in resident selection. Likewise, in a multicentric study, de Virgilio and colleagues [14] reported that those general surgery residents who were potentially at risk of failing the American Board of Surgery qualifying and certifying examinations could be identified early if they had a low Step 1 score. Additionally, Step 1 and Step 2 CK scores were correlated with better performance in the American Board of Emergency Medicine certifying examination, as reported in a multicenter study by Harmouche et al [15]. Further, in 2021, Filiberto et al [16], through a single-institution study of interns in all specialties, determined that step scores were significantly associated with better evaluations of intern performance by program directors.

## Rationale Behind the Scoring Change

The original purpose of the criterion-referenced examinations such as the USMLE, COMLEX, and MCCQE was not for sorting candidates for residency selection as done by the NEET-PG in India [6]. Rather, these exams were intended to be an assessment of the candidate’s competence for practice [2,3]. Thus, the USMLE Step 1 was primarily intended to deliver a pass/fail standard, but its scores in effect gradually became the major attribute being utilized by stakeholders in residency selection for decades [2]. Although the pass/fail standard (criterion-referencing) of the USMLE Step 1 was valid, reliable, and defensible, the same could not be said for its sorting function (norm-referencing). Thus, the primary rationale for the change was the attempt by licensing authorities to restore the USMLE Step 1 and COMLEX Level 1 to their original intended purpose [2]. Additionally, the overreliance on Step 1 as a screening tool often led students to prioritize this exam over the in-house medical curriculum at their respective institutions, with students reportedly showing less commitment to competencies not deemed “high yield” on the Step 1 exam [17-20]. A reported mismatch between their in-house curriculum and Step 1 preparation existed, in effect, a parallel curriculum [21-23]. Furthermore, students belonging to disadvantaged and underrepresented groups in medicine have historically and consistently scored lower on standardized exams, including the USMLE Step 1, stemming from a multitude of socioeconomic factors. Step 1 scores were therefore correlated with racial and demographic disparities, disproportionately impacting underrepresented minority candidates [24,25]. Additionally, several medical educators argued that Step 1 scores could not assess other crucial, yet subjective, competencies such as interpersonal skills and professionalism [26]. Thus, it was hoped that decreasing the reliance on Step 1 could help expand the holistic consideration of applicants from all backgrounds [26]. Although these limitations have been long-standing, little change had taken place in several years; therefore, when this change was announced, it was met with much surprise and concern.

## Impact of the USMLE Step 1 Scoring Change on Applicants

The impact of the USMLE Step 1 scoring change is likely to be enormous on all applicants, including US-MDs, US-DOs, and international medical graduates (IMGs), who may be either US citizen IMGs or non-US citizen IMGs, with the latter also known as foreign medical graduates. This impact was captured in several publications through surveys of residency program directors and applicants. However, these data should be interpreted with caution, as surveys are intrinsically limited by their response rates. If the response rate is 45%—the rate in the survey by Makhoul et al [27]—the survey’s bias is estimated to be 55% [28]. Response rates may also be related to representativeness, which further exacerbates this bias. Additional limitations include (1) a central tendency bias due to the use of a Likert scale [29], (2) potential selection bias of those with stronger opinions regarding the change, and (3) a lack of subgroup analysis of responding programs due to anonymity in reporting. Additionally, there are studies such as those done on the otorhinolaryngology residency application process [30], which have used different questionnaires; hence, findings from specialties may not be compared directly.

The major works that have been published on USMLE Step 1 scoring conversion are summarized in Table 1. Of note is the paper by Makhoul and colleagues [27] in the New England Journal of Medicine, with similar specialty-specific papers derived from data collected by this research group also published and widely available. The authors conducted a seminal survey of over 2000 program directors from various specialties, with responses providing clues regarding the impact of the scoring change on applicants [27]. Approximately 81% of the program directors felt that USMLE Step 2 CK would acquire more importance; therefore, it was perceived that the emphasis and anxiety had merely been shifted from Step 1 to Step 2 CK.

Exam-related anxiety is likely only to increase, as candidates now only have one chance to obtain a top score; this change has also removed the chance to demonstrate an improvement in scoring from Step 1 to Step 2 CK. A shift to a greater emphasis on performing well on Step 2 CK, which is taken later in medical school, has been hypothesized to adversely impact US-MD and US-DO performance in clinical rotations [31]. Importantly, given that IMGs have historically relied on high Step 1 scores for demonstrating their competitiveness in the residency match, the potential impact of this change cannot be overstated.

**Table 1:** Specialty-specific data and selected perspectives regarding the impact of United States Medical Licensing Examination Step 1 pass/fail conversion and the perceptions of various stakeholders.

Authors, year	Stakeholders	Journal name	Title of work
Makhoul et al [27], 2020	Program directors of all specialties	New England Journal of Medicine	Objective measures needed—program directors’ perspectives on a pass/fail USMLE^a^ Step 1
Mun et al [32], 2021	Program directors in internal medicine and orthopedics	BMC Medical Education	A comparison of orthopaedic surgery and internal medicine perceptions of USMLE Step 1 pass/fail scoring
Mun et al [33], 2021	Program directors in internal medicine	Medicine	Internal medicine residency program director perceptions of USMLE Step 1 pass/fail scoring: a cross-sectional survey
Ehrlich et al [34], 2021	US medical students	The American Surgeon	Implications of the United States Medical Licensing Examination Step 1 examination transition to pass/fail on medical students education and future career opportunities
Cangialosi et al [35], 2021	US medical students: perspective	Academic Medicine	Medical students’ reflections on the recent changes to the USMLE step exams
Gu et al [36], 2021	Program directors in orthopedics	Journal of the American Academy of Orthopedic Surgeons Global Research and Reviews	Effect of change in USMLE Step 1 grading on orthopaedic surgery applicants: a survey of orthopaedic surgery residency program directors
Asaad et al [37], 2021	Program directors in plastic surgery	Journal of Surgical Education	Applicant familiarity becomes the most important evaluation factor in USMLE Step I conversion to pass/fail: a survey of plastic surgery program directors
Lin et al [38], 2020	Program directors in plastic surgery	Plastic and Reconstructive Surgery Global Open	Implications of pass/fail Step 1 scoring: plastic surgery program director and applicant perspective
MacKinnon et al [29], 2021)	Program directors in radiology	American Radiology	Pass/fail USMLE Step 1 scoring—a radiology program director survey
Warren et al [39], 2021	Medical Twitter	Academic Medicine	#MedEd Twitter response to the USMLE Step 1 pass/fail score reporting announcement
Snyder et al [40], 2021	Residency applicants for neurosurgery	Journal of Neurosurgery	Applying to residency: survey of neurosurgical residency applicants on virtual recruitment during COVID-19
Romano et al [41], 2021	Neurosurgery program directors, program chairs, and program administrators	Journal of Neurosurgery	Optimizing the residency application process: insights from neurological surgery during the pandemic virtual application cycle
Mamidi et al [42], 2021	Program directors in otolaryngology	Annals of Otology, Rhinology, and Laryngology	Perceived impact of USMLE Step 1 score reporting to pass/fail on otolaryngology applicant selection
Chator et al [43], 2021	Program directors in physical medicine and rehabilitation	American Journal of Physical Medicine and Rehabilitation	Physical medicine and rehabilitation program directors’ perspectives on US Medical Licensing Examination Step 1 scoring changes
Glassman et al [44], 2021	Program directors in emergency medicine	The Western Journal of Emergency Medicine	Emergency medicine program directors’ perspectives on changes to Step 1 scoring: does it help or hurt applicants?
Patrinely et al [45], 2021	Program directors in dermatology	Cutis	USMLE Step 1 changes: dermatology program director perspectives and implications
Chisholm and Drolet [46], 2020	Program directors in urology	Urology	USMLE Step 1 scoring changes and the urology residency application process: program directors’ perspectives
Odei et al [47], 2020	Program directors in radiation oncology	Advances in Radiation Oncology	Potential implications of the new USMLE Step 1 pass/fail format for diversity within radiation oncology
Pontell et al [48], 2020	Program directors in general surgery, integrated vascular, integrated thoracic, and integrated plastic surgery	Journal of Surgical Education	The change of USMLE Step 1 to pass/fail: perspectives of the surgery program director
Erath et al [49], 2020	Program directors in anesthesia	Anesthesia and Analgesia	Program directors’ response to a pass/fail US Medical Licensing Examination Step 1
Huq et al [10], 2020	Program directors in neurosurgery	Journal of Neurosurgery	Perceived impact of USMLE Step 1 pass/fail scoring change on neurosurgery: program director survey
Ganesh Kumar et al [4], 2020	Program directors in neurosurgery	World Neurosurgery	Characterizing the effect of pass/fail US Medical Licensing Examination Step 1 scoring in neurosurgery: program directors’ perspectives
Manstein et al [50], 2021	Medical school deans	Plastic Surgery (Oakville, Ontario)	The upcoming pass/fail USMLE Step 1 score reporting: an impact assessment from medical school deans
Aziz et al [51], 2021	Program directors in general surgery	World Journal of Surgery	Selecting the next generation of surgeons: general surgery program directors and coordinators perspective on USMLE changes and holistic approach
Goshtasbi et al [30], 2021	Program directors in otolaryngology	Laryngoscope	The effects of pass/fail USMLE Step 1 scoring on the otolaryngology residency application process
Whaley et al [52], 2021	Pathology: perspective	Academic Pathology	Changes in USMLE Step 1 result reporting: a pass or fail for pathology programs?
Fiedler [53], 2021	Cardiothoracic surgery: perspective	Seminars in Thoracic and Cardiovascular Surgery	Commentary: USMLE Step 1 pass/fail = win/win for cardiothoracic surgery trainee selection
Rajesh et al [54], 2021	Residents (surgery): perspective	Journal of Surgical Education	Binary reporting of USMLE Step 1 scores: resident perspectives
Aggarwal [55], 2020	International medical graduates	Academic Radiology	USMLE Step 1 reported as pass/fail: did international medical graduates need a reform?
Wallach et al [56], 2020	Residents (internal medicine): perspective	Journal of Community Hospital Internal Medicine Perspectives	Internal medicine resident perspectives on scoring USMLE as pass/fail
Quesada et al [57], 2021	Otolaryngology residency applicants	OTO Open	Overemphasis of USMLE and its potential impact on diversity in otolaryngology
Ganesh Kumar et al [58], 2021	US medical students, residents	Journal of Graduate Medical Education	Comprehensive reform and greater equity in applying to residency-trainees' mixed responses to a pass/fail USMLE Step 1
Choudhary et al [59], 2021	Program directors in internal medicine	Journal of General Internal Medicine	Impact of pass/fail USMLE Step 1 scoring on the internal medicine residency application process: a program director survey
Pascarella [60], 2020	Clerkship director (perspective)	JAMA Surgery	USMLE Step 1 scoring system change to pass/fail-perspective of a clerkship director
Girard et al [61], 2021	US medical students	Journal of Surgical Education	US medical student perspectives on the impact of a pass/fail USMLE Step 1
Belovich et al [62], 2021	International association of medical science educators	Medical Science Educator	USMLE Step 1 is going to pass/fail, now what do we do?
Boulet and Pinsky [63], 2020	International medical graduates	Academic Medicine	Reporting a pass/fail outcome for USMLE Step 1: consequences and challenges for international medical graduates

^a^USMLE: United States Medical Licensing Examination.

A focus on research productivity was already a prominent requirement for a successful match into competitive specialties [64]. This may potentially further increase with the elimination of Step 1’s objective scoring. For IMGs in particular, this is anticipated to be a significant hurdle—medical student research opportunities remain abysmal in low- and lower-middle-income countries [65,66]. Even in institutions where research is encouraged, such as the authors’ medical schools, publishing is difficult with paywalls and publishing fees limiting integration into peer-reviewed indexed journals. In addition to research, an emphasis on letters of recommendation, Alpha Omega Alpha Honors Medical Society membership, and clerkship grades have been expected to become more pronounced in applications, particularly in competitive specialties. For example, according to a recent comparative study, orthopedics program directors were more likely to prioritize these factors when compared with internal medicine program directors [32]. This represents another limitation for IMGs and students outside of institutions with faculty whose letters carry weight in decision-making processes.

Rotating at outside institutions and subsequently obtaining a letter of recommendation from the said institution’s program director was considered instrumental in receiving invitations to competitive specialties such as dermatology, neurosurgery, orthopedics, and plastic surgery. Concerningly, with the move to pass/fail reporting and completing away rotations, colloquially called “audition rotations,” may become important even for noncompetitive specialties [67]. This may substantially increase the out-of-pocket costs for each medical student, further disadvantaging IMGs and financially less capable candidates [68].

Approximately 57% of the program directors reported that they would consider medical school prestige while evaluating candidates [27]. In the United States, Black medical schools and schools in Puerto Rico have historically produced the majority of African-American and Hispanic graduates; yet, these medical schools are rarely ranked highly [69]. Socioeconomic status and race are linked [70], and many of these disadvantaged students opt to attend more affordable institutions even if they are less prestigious. Thus, this scoring change could lead to a paradoxical worsening of the holistic review for these disadvantaged groups, leading to a further worsening of diversity across training programs [27].

In addition, a survey of plastic surgery program directors reported that personal prior knowledge of the applicant was one of the most important factors in evaluation [37]. This subjective metric of evaluation, often driven by multiple socioeconomic factors, may prove to be a less than ideal tool compared to objective measures, following the conversion of USMLE Step 1 to a pass/fail outcome. However, with the pressure to score well on standardized exams like USMLE Step 1 removed, or at the very least, delayed, to taking Step 2 CK, medical students may be able to pursue specialty interests via research early on, translating to better knowledge on clinical rotations and subsequent assessment metrics. They may be able to participate in more community activities and volunteering efforts. Additionally, it is possible that their mental health may improve, in the absence of a minimum score to aim for. Still, these perceived benefits should be contrasted with the aforementioned risks, as the net effect may still disadvantage underrepresented applicants as well as IMGs, particularly those aiming for competitive specialties [71].

Through direct and indirect effects, the Step 1 pass/fail change may likely impact IMGs adversely, especially foreign medical graduates, and may decrease foreign medical graduate representation in US residency positions. IMGs fill a crucial gap in the US health care system, serving groups of all backgrounds and in underserved areas [72,73]. IMGs constitute a significant proportion of the American physician workforce. In 2018, almost 25% of the residents and fellows were IMGs, even representing over 50% in some specialties [74]. They have provided and will continue to provide significant contributions toward addressing the physician gap in the United States. In neurology, for example, the physician workforce gap is projected to increase by 18% by 2025 [73,75,76]. Interestingly, after accounting for physician and practice characteristics, IMGs deliver medical care more often than US graduates for complex patients, with lower mortality rates for older Medicare patients, and reports indicate no differences in readmission rates while accounting for hospital indices, patient characteristics, and socioeconomic status [77]. Given the high-quality care provided by IMGs and the dependence of the American health care system on IMG service for sustenance, the change of USMLE Step 1 to a pass/fail outcome has, thus yet, unclear but far-reaching consequences for IMGs and their matching into primary care specialties.

An important demographic to also consider includes DO candidates. Their match success rates, particularly in competitive specialties, have traditionally been far worse than their MD counterparts [78]. A standardized DO candidate will write the COMLEX Levels 1, 2, and 3, typically taking USMLE Step 1 in tandem with COMLEX Level 1 for consideration in the residency match. In addition to the loss of the opportunity to becoming a more competitive applicant with a high USMLE Step 1 score, DO students may now need to prepare for USMLE Step 2 CK in tandem with COMLEX Level 2 following their clinical rotations. However, most osteopathic programs maintain a traditional curricular calendar with clinical rotations ending in June, thus leaving DO applicants without protected time to adequately prepare for USMLE Step 2 CK, COMLEX Level 2, and subinternships/away rotations, further exacerbating the residency match for osteopathic medical students [62,79].

## Impact of the USMLE Step 1 Scoring Change on Educators

The impact of the Step 1 scoring change on educators, particularly program directors, will likely be multifaceted. Each year, candidacy to residency programs has steadily risen, with over 40,000 applicants in 2020 [9]. Similarly, the number of applications submitted per applicant has increased, forcing program directors to use Step 1 scores as a screening tool. This is especially true for IMGs in internal medicine—the specialty taking the largest number of IMGs. In 2019, IMGs submitted an average of 98 applications [80] compared to an average of 35 applications by US-MDs/DOs [63], making Step 1 to be the one reliable metric for program directors to screen candidates. Considering this, only 15.3% of all program directors surveyed by Makhoul et al [27] agreed with the USMLE Step 1 scoring change. In fact, the Association of Program Directors in Radiology announced their opposition to the USMLE Step 1 pass/fail format in August 2019 [81]. Importantly, although the InCUS meeting was supposed to represent all stakeholders, it was reported that leaders from the Graduate Medical Education community felt underrepresented in this decision-making process [82]. For educators, the increasing number of applications conflicts with the holistic application screening, leading to greater use of objective measures, with USMLE Step 2 CK likely becoming the preferred screening tool in lieu of Step 1 after the pass/fail change. Over 77% of the program directors indicated their belief that this change would make it more difficult to objectively compare candidates [27]. In some specialties such as neurosurgery, Step 1 scores have been shown to correlate with neurosurgery board exam scores [83], and similarly, in obstetrics and gynecology, USMLE performance was correlated with that of resident evaluation exams [84]. In the otorhinolaryngology board exam [30], underperforming (score<210) was linked to a higher chance of not passing board exams. Regardless of the debate surrounding their predictive utility [11], underperforming in specialty boards incurs fines on programs; therefore, these potential correlations were valuable for program directors.

It remains to be seen how medical institutions will adapt their curricula to the USMLE Step 1 scoring change. US medical schools may change their calendar to allow students to take Step 2 CK earlier, with a clear advantage for candidates from programs with an accelerated preclinical curriculum. Some authors have pointed out that this change may allow medical schools more curricular flexibility and take courses on topics not related to Step 1 but those useful for medical practice [26]. For many IMGs whose schools follow a 6-year schedule with inflexible preclinical curricula designed by national authorities in response to their national need, modifications in response to a US exam–related change are unlikely. One noteworthy concern for program directors is a decrease in the basic science knowledge, which forms the bulk of the Step 1 curriculum of medical graduates [49]. For specialties like anesthesia [49], which utilize conceptual frameworks heavily from basic sciences, this unintended consequence could have potential far-reaching, but currently little understood, impact.

After the scoring conversion, it is anticipated that program directors may now have to more closely look at Medical Student Performance Evaluations (MSPEs) or dean’s letters. Medical schools in the United States have continued to move from a ranked or scored evaluation to a pass/fail curriculum or similar broad categories [46,85]. Although dean’s letters are often lengthy and time-consuming to evaluate, they offer detailed insight into a candidate’s suitability for a particular residency position. However, because the evaluation criteria for international medical schools vary widely, MSPEs of IMGs have historically carried a significant degree of heterogeneity, with their distinguishing capability often questionable.

Taken together, the conversion of USMLE Step 1 from a 3-digit numeric score to reporting a pass/fail outcome alone may leave program directors with a challenging task for adequate and holistic, yet time-bound, evaluation of applicants. Efforts are being made through the introduction of Preference Signaling and ERAS Supplemental Application in the residency match to provide for a more holistic review and to ensure a better match between programs and applicants. Odei and colleagues [47] suggested the consideration of 7 components for residency candidates: research achievements, academic scores, commitment to the field, demonstrated compassion, demonstrated leadership, interpersonal skills, and diversity of life experiences [47]. Similarly, Makhoul et al [27] suggested a composite score consisting of shelf exam results in the major clinical subjects as an objective measure [27]—this may offset the bias toward Step 2 CK [86].

## Recommendations for Residency Applicants

To further break down the path to a competitive application to any residency program, at the beginning of their medical school career, often referred to as the preclinical or preclerkship years, junior doctors should seek mentorship and advice regarding various avenues available prior to residency application. Concurrently, they should seek shadowing and research opportunities with faculty members at their respective institutions, if possible, or at nearby medical programs if they do not have a home program [87-89]. As with every field, attaining familiarity with faculty members in the desired discipline may facilitate opportunities for increased success, which may be reflected through research (published abstracts, peer-reviewed manuscripts, textbook chapters, etc), strong letters of recommendation, additional biomedical honors (eg, research paper prizes), time devoted to specialty (summer research, research electives, away rotations in the specialty, etc), and attendance at key networking events (conferences, continued medical education accredited events, grand rounds, etc). Utilizing these opportunities may help applicants aiming for competitive residency programs. Additionally, given the increasing conversion of standardized national and international examinations to pass/fail, medical students should ensure securing the highest marks in every facet of their application that still provides scores or grades, such as preclinical exams, clerkships, or subinternships, COMLEX Level 2, and USMLE Step 2 CK. Importantly, securing protected research time becomes paramount to differentiate one’s application for residency, and medical students, including IMGs, considering a competitive match ought to consider taking one or more years dedicated solely to increasing their research productivity [90]. With regard to research productivity, in recent years, especially for competitive specialties, the average number of research experiences has increased, with some using the term “arms race” to describe this [64]. With the Step 1 scoring change, such experiences may only acquire potentially heightened importance. This is especially true for medical students from institutions known to have prolific research output—programs may have heightened expectations [10,91]. Of note, taking time out of clinical occupation for research may necessitate a serious commitment to readjusting to the demands of a clinical medical curriculum to maintain high academic marks, and students must perform effective cost-benefit analyses before every decision. Still, the combination of a stellar academic record, outstanding letters of recommendation, effective networking, and demonstrated interest in research may be more than sufficient for obtaining a competitive residency position. We have summarized some key official resources that applicants may refer to in Table 2 [2,68,92-95].

**Table 2:** Key official resources for applicants.

Organization, work			Remarks
**National Residency Matching Program**			
	Main residency match data and reports: 2022 [92]	A detailed report of characteristics of matched and unmatched applicants, allowing students to get a rough idea of what they need to do to enroll into their specialty of choice	
	Charting outcomes for the match: international medical graduates, 2020 and 2022 [92,93]	Data specific to international medical graduates	
	Interactive charting outcomes for the match [94]	Granular database of individualized charting outcomes, which permits candidates to assess their chances overall by inputting their personal attributes	
**United States Medical Licensing Examination**			
	Summary report and preliminary recommendations from the Invitational Conference on United States Medical Licensing Examination Scoring, March 11-12, 2019 [2]	A detailed assessment of the rationale and process behind the scoring change. The website also provides a list of references with a summary of the papers cited.	
	United States Medical Licensing Examination Step 1, frequently asked questions [95]	Frequently asked questions regarding the USMLE^a^	

^a^USMLE: United States Medical Licensing Examination.

## Conclusions

Given the unique history of USMLE Step 1 in the US residency selection process and the score’s correlation with future performance in specialty board–certifying examinations, this scoring change is predicted to significantly impact all stakeholders involved in residency selection. Empirical data on the impact of this change will likely only be available from the literature emerging after the conclusion of Match 2023 and potentially even Match 2024. However, some comprehension may be reached from reviewing the surveys and perspectives coauthored by applicants, program directors, leadership of professional organizations, among others. For aspiring physicians pursuing a US residency, considering the progressive conversion of both medical school and national examinations from a scored outcome to pass/fail, the focus should be made on building a holistic application for the specialty of choice. Candidates aiming to secure competitive residency positions may take additional steps, including, but not limited to, engaging in specialty-specific research opportunities, networking with candidates at every stage of their medical careers, and becoming involved in organized groups around the world.

## Abbreviations

CK: clinical knowledge
COMLEX: Comprehensive Osteopathic Medical Licensing Examination
DO: Doctor of Osteopathic Medicine
ECFMG: Educational Commission for Foreign Medical Graduates
FSMB: Federation of State Medical Boards
IMG: international medical graduate
InCUS: Invitational Conference on United States Medical Licensing Examination Scoring
MCCQE: Medical Council of Canada Qualifying Examination
MD: Doctor of Medicine
MSPE: Medical Student Performance Evaluation
NBME: National Board of Medical Examiners
NEET-PG: National Eligibility cum Entrance Test for Post-Graduation
USMLE: United States Medical Licensing Examination
